# Supported Employment, Participation at Work, and Peer Support: A Qualitative, Participatory Case Study Report of the Geesthacht Model

**DOI:** 10.3389/fpsyt.2021.634080

**Published:** 2021-04-23

**Authors:** Sebastian von Peter, Lena Göppert, Jenny Ziegenhagen, Timo Beeker, Rosa Glück, Birte Groth, Uwe Groß, Arne Reinholdt, Robin Boerma, Matthias Heißler, Juri Habicht, Julian Schwarz

**Affiliations:** ^1^Department of Psychiatry and Psychotherapy, Brandenburg Medical School, Immanuel Albertinen Hospital Rüdersdorf, Rüdersdorf, Germany; ^2^ExPEERienced – Experience With Mental Health Crises – Registered Non-Profit Organization, Berlin, Germany; ^3^Department of Psychiatry, Psychotherapy and Psychosomatics, Johanniter Hospital Geesthacht, Geesthacht, Germany

**Keywords:** peer support, occupational therapy, UN CRPD, mental health, coproduction, participatory research, power dynamics, vocational rehabilitation

## Abstract

**Background:** For people who have experienced mental health crises or psychosocial disabilities, it is considerably more difficult to receive support to participate in work on an equal basis with others. In the town of Geesthacht, in Northern Germany, an integrative care network was implemented that allows for acute psychiatric treatment as well as participation in work and activities. This paper aims to explore the principles, advantages, and challenges of this innovative project.

**Methodology:** Within the context of a participatory and collaborative process evaluation of a prospective controlled cohort study (PsychCare), researchers with and without experiential expertise conducted expert interviews and focus groups to evaluate the experiences of 37 employees, with and without lived experience, from various institutions associated with this care network. The data was analyzed using qualitative content analysis.

**Results:** It was the change from financial compensation paid on a daily basis to a global treatment budget that allowed for a significant reduction of hospital beds in Geesthacht and freed up resources to implement a complex care network. Since then, various possibilities for participation, work, and activities for former service users, some of which are compensated financially, have been made available. These developments now allow for a less bureaucratic and often smooth transition from being a service user to involvement in participatory activities in the role of a peer, which is frequently perceived to be empowering and beneficial by participants with lived experience. At the same time, this care model has led to multiple role conflicts and different challenges for all parties involved.

**Conclusion:** This innovative project in Geesthacht demonstrates the multifaceted potential of a global treatment budget system in the field of mental health care. To address certain downsides of the Geesthacht model, further development is necessary.

## Background

The United Nations Convention on the Rights of Persons with Disabilities (CRPD) calls for a different approach in terms of how we think about and act together with people with lived experience of mental health crises and disabilities on both a societal and institutional level. In doing so, the Convention upholds their legal entitlement to equal opportunities and equal participation in all areas of life as a central tenet. This principle gives concrete expression to long-standing, human rights-focused debates that all human beings may have the same rights, independent from their capabilities and disabilities (1). In particular, the CRPD aims at ensuring equal rights and participation in education, leisure and work activities, housing, and mobility. A self-determined and autonomous life is intended for everyone, in accordance with their own will and preferences.

In the field of mental health, many of these principles resonate well with long-debated concepts that originated within the social movement of survivors of psychiatric services, who are largely critical of the psychiatric care system. The notion of “recovery” has now been implemented in various health policy programs, implying self-determination, personal growth, participation, and control of one's own living conditions (2). The concept of “empowerment” has equally been coopted by various psychiatric institutions or stakeholders, yet its original meaning designated self-determination and choice of those people who should be the focus of care, both of which are indispensable conditions for effective participation.

To meet these demands and enable meaningful participation, extensive changes to the care system are necessary. Many changes did occur during the de-institutionalization phase that took place in a number of countries in the 1980's. However, the effects of this did not live up to aspirations. Although large institutions were closed in many places, the openness of communities and the possibilities to reintegrate so-called inmates within the communities remained very limited. While there are more recent draft laws aimed at fostering participation (3, 4), the majority of people with psychosocial or intellectual disabilities in Germany are still employed in segregated workplaces. The increasing number of beds in nursing homes and hospitals almost everywhere in Germany, among other structural mechanisms of exclusion, leads to a situation in which these people struggle to establish their existence independently from institutions and find inclusion in society.

This paper presents part of the results from a participatory process evaluation that took place within the context of a larger prospective controlled cohort study (PsychCare) (5). Various opportunities for people with lived experience of mental health crises and disabilities in a psychiatric care network in the town of Geesthacht in Northern Germany to participate in work and activities were explored. These opportunities have evolved from a longer process of reorganizing psychiatric care in Geesthacht with the aim to better assist people with lived experience in leading a more autonomous and self-determined life rather than assuming the identity of a help-seeking, disabled person. This paper focuses on only one segment of that transformative process - the gradual development of more prospects for participation in the fields of work and activities. This aspect was chosen, as it reflects the main focus of the work carried out in Geesthacht over the past years and demonstrates how these changes are presently perceived and evaluated by the people involved. In the discussion, the participation model in Geesthacht is considered critically, especially in relation to the question as to whether or not it meets CRPD-compliant criteria. To evaluate the Geesthacht model on its own terms, we have deliberately avoided comparing it with other models or insight of the international literature, instead focusing our discussion on its inherent tensions and ambivalences.

## Materials and Methods

### A Note on Language Used

This text was jointly developed by people with and without lived experience with the psychiatric care system, mental crises and disabilities, and recovery from them. This is one reason, among others, why it is important to us to handle language carefully. We purposely omitted terms such as “illness” or “disorder,” instead using the language of the CRPD, such as the term “mental health crises” and “psychosocial disabilities.”

Considering the variety of possibilities for work, activities, and participation in Geesthacht, it was difficult for us to find a single term to describe all the areas of involvement and roles connected to them. Throughout this report, different types of work, activities and/or formal employment are dealt with, such as paid employment on the basis of various forms of financial compensation and contracts (the wages ranging from usual market levels until small, additional forms of income), unpaid, volunteer work (by people with and without lived experience), or therapeutic activities (in the sense of occupational therapy, supported employment or more traditional forms of work therapy). To demarcate these different forms from each other at least to some extent, we refer in the following to paid work as “employment” and to unpaid work as “activities.” The gray area of supported employment [covering both place and train (individual placement and support) and train and place (in the sense of vocational training) versions] or more traditional forms of work therapy, in which therapeutic support may transition into employment will be indicated accordingly. All opportunities that are mentioned are offered by the same or different institutions, meaning that one and the same institution may offer one or more types of work and/ or activities.

The overarching term “employees with lived experience” is used to refer to the group of people that work in the role of former service users in various ways at the Geesthacht care network facilities. This term does not indicate if these people receive financial compensation for their work. Personal experience of mental crises or disabilities is not always fundamental to their work or activities. If so, we refer in this case to the terms “peer” and “peer support.” Yet, these terms do not imply that in all cases a specialized education, such as Intentional Peer Support, had preceded the support work. In some case, the concerned persons have undergone an Ex(peerienced) IN(volvement) training (6), or another form that has been developed and offered by peers, working in the Geesthacht care network, in some cases, the employees with lived experience offer support without any previous peer training, which is due to the historical development of the project.

### Care Network in Geesthacht

The Psychiatric Department at the Johanniter Hospital in Geesthacht was founded in 1997 and is in charge of providing care for the Duchy of Lauenburg county, a catchment area of 195,000 inhabitants to the east of Hamburg and south of Lübeck. The Duchy of Lauenburg is a rural area with 156 inhabitants per km^2^.

In 1996, even before the Psychiatric Department was established, a non-profit organization started to provide work opportunities and activities for people with psychosocial disabilities. Since 2007, the psychiatric hospital's structure has been fundamentally reorganized, first on the basis of a regional psychiatric budget system, and later with the change to a “global treatment budget” pursuant to §64b of the German Social Code, Book V (7). The number of hospital beds has been progressively reduced from 50 to 18, leading to one of the lowest rate of psychiatric hospital beds per inhabitant in Europe: On average there are 69 beds in the EU (Germany: 128) for psychiatric care per 100,000 inhabitants. In the Duchy of Lauenburg this value is just under 11 / 100,000 (8). Resources were released through this reorganization that were reinvested in various forms of home treatment, housing therapy (“Housing First”) and supported employment, as well as to support cross-sectoral cooperation. From the very beginning, occupational therapy taking place in outpatient practices was included in the budgets, as it had been traditionally used heavily in this catchment area prior to the years of reorganization in Geesthacht.

A more detailed description of these reorganization processes cannot be given in this paper due to limited space (9). But as a result of this reorganization, a complex and comprehensive network of institutions inside and outside of the hospital was established, spanning across different treatment sectors, and including various non-profit organizations, volunteer networks, cooperating doctor's practices, outpatient centers, home treatment teams, and forms of peer-organized crisis centers and respites. Developing this network enabled more continuous, flexible, and empowering treatment and support across the sectors and within the service users' home environment. Even more noteworthy is the fact that this network was created and built up at a time when neither the CRPD, nor any of the concepts of empowerment, supported employment, housing first, recovery or peer support had been applied on a larger scale, at least in Germany.

Table 1 lists the actors and institutions cooperating with the Geesthacht model. Some of the participating partners receive payment for their services, either through health care funding (according to the German Social Code, Books V and XI) or *via* funds from rehabilitation programs (according to the German Social Code, Books IX and XII). Some collaborating institutions generate their own income or finance work based on donations. In the past few years, the global treatment budget was somewhat flexible, making transfers of funding between the institutions and sectors involved possible. Today, however, fewer of these options exist due to structural reasons (see discussion). Consequently, the cooperation between the actors and institutions involved has become more limited, thus hampering the continuity and flexibility of the related services.

**Table 1:** Overview of the main institutions (and provided services) that cooperate with the Geesthacht model.

**Institution / facility**	**Provided services**
Practices for occupational therapy	- Social work - Occupational Therapy
Psychiatric hospital care	- Psychiatric inpatient - Day-/Nightpatient - Outpatient - Outreach Care
Living support	- Private living - Crisis respites - Home care groups - Housing First
Social participation facilities	- Day centers - Laundromat Café - Charity shop - Second-hand market - Agriculture and Gardening
“ABC-Team”	- Connecting Stakeholders and institutions, - “Job placement”
Outreach nursing care	- Psychiatric Nursing Care
Vocational support facilities	- Hospital Logistics - Facility Management - Patient Shuttle - Catering services

*The presentation is simplified; a more comprehensive version can be found in the Supplementary Table 1*.

It would be impossible to describe here all of the developments in Geesthacht in detail over the last 13 years. Therefore, we have chosen not to present a more nuanced description of the advancements made in the fields of housing and home treatment in this manuscript and, instead to focus on the fields of work and participation. These advancements have led to various opportunities for work and participation for people with lived experience of mental crises and disabilities since 2007, both inside of the hospital and in the cooperating institutions in Geesthacht. This emphasis on work and activities in Geesthacht originated conceptually during the period of de-institutionalization of psychiatric care in the 1970's (10). Some of the initiators of the more recent reorganization processes in Geesthacht were also part of the de-institutionalization movement at the time and among other initiatives, contributed to the closure of one of the largest psychiatric asylums in Gütersloh/ Germany. Many years later, the concept of peer support has become increasingly popular (11, 12), and was also adopted in Geesthacht, together with the concepts of recovery and empowerment that over time developed into some of the guiding principles of the care network as it is practiced now. This origin of the Geesthacht model in the 1970's and its evolution over time has led to certain conceptual tensions, for instance understanding work as therapy or as a right, tension that will be discussed to be productive further below.

### Study Design

This exploration is part of the multicenter study “PsychCare” (2017–2021) that included a participatory process-evaluation of Flexible and Integrative Treatment (FIT) models provided for by law (§64b of the German Social Code, Book V) in Germany (5). In total, a cohort of 10 FIT and 10 control departments were included in the study, although the results from the evaluation of only one region are included in this paper. The process-evaluation aimed at the assessment of the level of implementation using operationalized program components and statistical parameters (13, 14). In Geesthacht, a high level of implementation was found. In addition, this region showed several structural idiosyncrasies that were not sufficiently captured through this multicenter study. This was taken as an opportunity to study in further detail the range of services available in Geesthacht.

A qualitative methodology was chosen as best suited to meet the needs of this exploratory research (11). A collaborative approach was used throughout the process evaluation: researchers with their own experiences with the psychiatric and mental health care system, mental crises and disabilities and recovery, and researchers without these experiences worked together (15). This approach also fits the Geesthacht model that aims to strengthen the collaboration of employees with and without lived experience. The present manuscript only refers to some of the results that came out of this collaborative research process. Other research results will be published elsewhere (16), the same as some of our reflections on our research process as a team.^1^

### Data Collection

Our study was based on two qualitative surveys. In May 2018, 10 semi-structured expert interviews were conducted by SvP and JS with senior staff from the hospital and some cooperating institutions in Geesthacht to explore the implementation of the FIT model provided for in §64b of the German Social Code, Book V (17). Participants were selected using a snowball sampling (18). During a two-day field visit, all institutions involved in the care network of the region were visited, their network relations were mapped, and this visualization was coordinated (Table 1).

Then in February 2019, a second survey was conducted by a team of five researchers (RG, JZ, LG, TB, and SvP)—among them three with lived experience. In total, two group discussions and 14 individual interviews were conducted. Study participants were selected using a convenience sampling and those with lived experience were interviewed by peer-researchers to allow for a better exchange (19). The group discussions aimed at collecting data on the cooperation among the various actors, and at creating a space for diverging viewpoints (15). One of these discussions involved clinical staff of the hospital in Geesthacht (*n* = 5; employees without lived experience), while the second group was constituted exclusively of employees with lived experience (*n* = 8). In addition, individual interviews were conducted with employees with lived experience (*n* = 7), with non-professional employees without lived experience and no formal training in psychosocial care (*n* = 2), as well as with employees without lived experience (*n* = 5). The latter included the current and former heads of the Department of Psychiatry and Psychotherapy in Geesthacht.

A semi-structured interview guide was used for the group discussions and interviews. This guide included the following main topics: (1) operating principles and prerequisites of the Geesthacht model regarding participation in work and activities, (2) perceived effects of the collaboration between employees with and without lived experiences, (3) different opportunities for the employees with lived experience to work and the available forms of compensation. The relevant parts of the guide can be found online in the Supplementary Table 2. Additional questions focused on the home treatment system in Geesthacht and on the Housing First program but are not listed in the supplement due to the focus of this paper. All surveys were recorded digitally, transcribed, and then anonymized. All participants who were interviewed gave their informed written consent. The study was approved by the Ethics Committee of the Brandenburg Medical Association (Landesärztekammer Brandenburg) [2017, No. S7(a)] and by the Ethics Committees of the Federal States the participating hospitals were located in.

### Data Analysis

The transcripts were evaluated using qualitative content analysis (20) to reduce the data and match the collected material with meaningful categories. As part of the collaborative approach and to meet the quality criteria of qualitative research, all members of the research team coded individually. The codes were then merged in the group, whereby individually developed codes were coordinated and added to build up a coherent coding tree. The preliminary results, as well as this paper in a German version were discussed and validated with the interviewees in Geesthacht (18).

## Results

Only results related to the areas of work and activities are listed and discussed with the objective of making this paper easier to understand and accessible. As mentioned above, we have omitted a discussion of other innovations in the fields of treatment and housing that have nevertheless also occurred in Geesthacht.

In total, 37 persons participated in our evaluation. Roughly half of them (*n* = 19; 51.1%) called themselves employees with lived experience. Among them, 81.1% had several years of experience as users of the mental health care system in the study region. 64.8% (*n* = 24) of all study participants were female. The majority of the participants without lived experience worked in executive positions. To guarantee the anonymity of the participants, a more detailed presentation of the sociodemographic characteristics was omitted in this paper.

In the interest of readers, the results are presented in two parts: Part I explains how participation in the fields of work and activities is ensured in Geesthacht, describing the allocation of tasks, and which prerequisites are necessary. Part II provides the participants' experiences and evaluations. This division into two parts does not represent the multiple, recursive and circular connections of our analytical codes that made this evaluation very elaborate and interesting. It also reduces the lived complexity in Geesthacht, where different forms of participation are interlaced with each other. In the following, citations are marked either by E (employee without lived experience) or ELE (employee with lived experience).

## Part I. How the Geesthacht Model Operates

Opportunities for work and activities in Geesthacht are described in this section using both citations and figures, whereas the evaluations of the participants are reserved for part 2.

### How Service Users Become Employees

The form of activity or work varies depending on both clinical requirements and the person's needs and capabilities. Often, employees without lived experience are the ones who start looking for activities or work for persons with lived experience:

*“It's a bit like placing a volunteer or something like that. Together with the people that are interested, you simply see [what's possible].”* (E)

Sometimes, opportunities for activities or work evolve from a definite situation, or people find out from others that it is possible to work in the mental health care system as a (former) service user:

*“Being on the ward, we went out shopping and cooked. Even after their discharge, service users sometimes said: ‘Come on, let me continue to help out.’”* (ELE)

*“Naturally, it has to do with how people progressively recover, […] how people hear about it somehow by word of mouth…” (E)*

Finally, there is a networking group that acts as a hub for work placements and organizes various forms of participation:

*“[The group on Tuesday] is kind of a group meeting […] if someone says, ‘I want to participate!’, the first step would be to join us in one of these meetings. It is not like ‘oh, now we have to make a contract’ […] at some point you notice if it works out or not. […] It's not so official, you don't have to report or send an application to participate, but first you have to […] get to know each other a little*. (ELE)”

### Growing Within the System

Different opportunities for work and activities may exist and alternate for former service users. These opportunities involve different roles and kinds of work that may lead to increasing autonomy in accomplishing tasks and also be financially rewarding, when possible:

*“For example it may have started [with cooking]: ‘Back then, it was good but it's not really my thing anymore. ‘In that case, they may go on to do other tasks: ‘I have become pretty stable. […] I want to be part of the peer advisory group. ’”* (E)

The transitions between first the use of therapeutic services (in the role of a user/patient), then to voluntary forms of participation, and finally, to paid employment are often smooth:

*“This lady […] made coffee in the shelter for many years [as part of outpatient occupational therapy]. One day, she worked as the supervisor for the Saturday occupational therapy sessions. For many years now, she has been engaged in peer support.”* (E)

The conditions of expenditure of the regional psychiatric budget allowed for greater flexibility in terms of use and transiting between these stages of the program. The following citation illustrates, for instance, how resources from the regional budget were used to establish an outpatient care service under the supervision of an employee with lived experience:

*“There is this nurse manager who was suffering from depression […] who says: ‘I want to do something useful again […]’ ‘and the others said: ‘Okay, that is fine, we need a new nursing service.’ And she got the financial support to build up a nursing service.”* (E)

**Figure 1:**
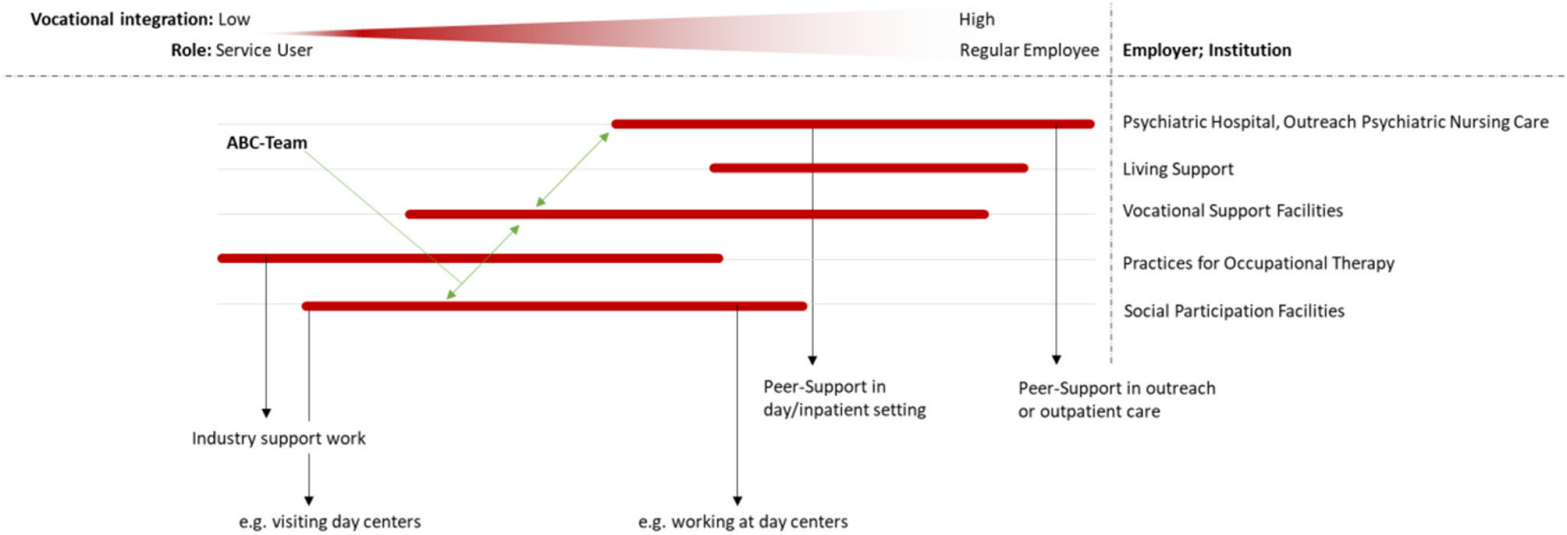
The opportunities to participate in the fields of work and activities are shown as a gradient in relation to the implementation of work-related participation and the different roles (i.e., service user to employee, for example in the field of peer support). The ABC team makes a flexible change possible between workplaces and tasks, occupational support facilities are regulated by the underlying financial structure in each case.

The gradual opportunities for work and activities are displayed in Figure 1.

### Forms of Employment and Compensation

The opportunities for participation and related financial rewards, if available, can only be addressed descriptively due to limited space. A more detailed description can be found in the Supplementary Table 1. As shown above, in Geesthacht, a plethora of different activities for employees with lived experience exist that are continuously evolving. Many of these activities do not fall within the definition of peer support and involve areas where experiential expertise is not directly necessary or applied. At the same time, however, the total share of employees with lived experience has increased through the availability of these different types of work and activities.

The various forms of such work and activities include: employment in hospital warehouses, hospital bed maintenance, working in transportation services, or being a member of the peer support team or a social counselor, including in the context of the various home treatment teams. The tasks are carried out in the service users' home environments, in clinical-therapeutic settings (hospitals wards or in the outpatient institutions of occupational therapy), or in the management departments (administrative unit or in the cleaning and transportation services) (see Table 1). The activities that are offered require and encourage different levels of autonomy and self-determination and can therefore be helpful at different stages of the recovery process.

The organizational framework governing many of these opportunities for participation consists of various models of collaboration, employment, or payment. Due to the lack of clear funding structures for participation in Germany, creative ways had to be found in Geesthacht to make financial compensation possible. Most employees with lived experience begin their activities while still being a service user, although their situation may vary (e.g., inpatient, day-care hospital, and outpatient). Initially, they often start with activities as part of work or occupational therapy. Subsequently, they work as volunteers and later in positions that could be called a type of “supported employment.” Different opportunities arise from these situations and can lead to voluntary forms of participation, paid work, or employment. The underlying funding models range from financial compensation to allowances using resources from the global budget, payments equivalent to “additional income”^2^ or a so-called “mini-job,”^3^ or financial resources which are provided by the social insurance system and can be paid out to support insured patients (e.g., through psychiatric nursing or everyday support) or through personal budgets allocated. During the initial phase of the project, many of these resources were administered by the relevant institutions and could be managed jointly, making it possible to hire a number of employees with lived experience. The transition from a regional budget to the global treatment budget in 2013 resulted in lesser financial flexibility.

### Philosophy and Mindset

The system described in this paper requires a certain way of thinking and acting on behalf of the institutions involved. The following section consists primarily of thoughts and citations from executive staff (all of them without lived experience), therefore presenting a potentially idealized (and idealistic) image of the Geesthacht model.

As mentioned in the beginning, the initiative to make these changes in Geesthacht was based on genuine values of traditional forms of work therapy. According to these values, work has a therapeutic benefit and therefore plays an important role in every person's life:

*“The idea has always been: everyone wants to be needed, wants to do something useful. Especially people with no opportunities on the regular job market.”* (E)

*“For me, it is like some sort of art therapy. I get to go out and get to do something useful.”* (ELE)

A second principle in Geesthacht is orientation based on the person's needs. All processes are geared to the perspective of what the person explains they need and want. Based on this idea, participation is implemented in daily routines allowing for “organic growth,” which leaves sufficient space for spontaneity, chances and changes:

*“I cannot say that that he is or is not able to do that, the people [employees with lived experience] have to know that for themselves.”* (E)

*“You must allow room for chance opportunities.”* (E)

Third, in many interviews the phrase “let them do it themselves” was articulated. This includes encouraging others and oneself to relinquish control and to rely on autonomy and self-responsibility:

*“[…] that let go, let the other person do it, and encourage him to take over tasks, for me that was the beginning of it all […]”* (E)

*“It is important that you believe in people. And that you do not leave them alone.”* (E)

This “let them do it themselves” attitude is common in Gesthaacht, even during treatment. This attitude is important for pragmatic reasons. There were situations, when some of the services in Geesthacht could not have been run without the support of peers involved, or still do not, either due to a shortage of employees or because only employees with lived experience have the necessary expertise:

*“We had no choice but to ask our patients to support us and take over tasks.”* (E)

*“This used to be some kind of a self-help ward, you could say. There were some employees but sometimes not enough, so service users sometimes had to organize themselves somehow.”* (ELE)

*“Yes, we used them purposefully to enter into contact with particular patients when we thought we were not able to reach the person.”* (E)

Furthermore, employees with lived experience often take over unpopular jobs like bureaucracy, cleaning, and logistics:

*“All these bureaucratic [jobs], the jobs that [they] are happy to hand over [to the peers].”* (ELE)

*“There is no additional pay for working on Saturdays. That means employees are happy when other people can take over this shift, it is give and take and has advantages for everyone.”* (ELE)

## Part II: How the Geesthacht Model Is Perceived and Evaluated by the Participants

This section reviews the evaluations and experiences of the persons that used the opportunity to participate in the fields of work and activities. We primarily present citations by operative employees (vs. executive staff reported on in section Philosophy and Mindset above). Although the experiences and evaluations of both groups coincide to some extent, fundamental discrepancies became apparent as well which merit description.

### Role Conflicts

For many employees with lived experience, questions related to their role and the changes to their role were central. The following citations illustrate how they felt about the position they have within the team, the forms of knowledge they were expected to have or were able to apply, and the significance of their experience within the teams or the institution:

*“What is the position of peers within the team? This all remains part of an ongoing discussion and debatable.”* (ELE)

To date, no clear description of the role or expectations of employees with lived experience has been established. Similarly, no well-defined concept of experiential knowledge or expertise has been formalized:

*“So far, a clear role description for peer support is lacking.”* (E)

*“What makes it special, what is the unique characteristic of the activities and contributions of people with lived experience? And what is the impact when these practices become institutionalized?”* (ELE)

Given the high level of ambivalence and many role changes, the lack of distinct role descriptions was problematic for some of the interviewees:

*“Sometimes, it was really difficult for me to distinguish - am I the patient now or do I work as part of the staff? That gave me a few headaches. […] It was too much.”* (ELE)

*“It went so far that my boss was simultaneously my therapist […] we framed this with the famous hat trick: ‘Well, now I'm wearing the hat of the therapist, or of the client, or of the employee.’”* (ELE)

*“…being both at the same time, for me, it was pretty simple. Back then, when I was really sick, I lived but also worked in this housing project. It was visible for everyone. […] But that's the way it is in real life - I can feel deathly ill and at the same time I can still help you. The barrier is artificial, it has been created by psychiatry. My hope is that, if people with lived experience gain power, this naturalness of giving and taking will be restored.”* (ELE)

### Lack of Financial Compensation Structures and Related Risks of Exploitation

As described in section Forms of Employment and Compensation, a comprehensive, flexible and person-centered funding system is lacking in Germany that would allow for participation of service users in the life of the community. The Geesthacht model tries to bridge this gap through creative ways of providing compensation, although not always successfully. Consequently, we are aware of the risk that the Geesthacht model could lead to exploitation of employees with lived experience. They do important work but are paid only partially or not at all for it:

*“By now I think that it should be paid […]. Initially, I thought: ‘Well, just give it a try, you'll get [money] at some point when you are really a part of it’.”* (ELE)

Likewise, a number of employees with lived experience complained that the financial compensation for their work was insufficient. Moreover, payments varied significantly from person to person. This is mainly due to structural reasons, yet often caused dissatisfaction:

*“You have to know that peers are far from being able to make a living [with that activity].”* (ELE)

*“Everyone at this table receives a different amount or no payment at all for the same amount of work. […] People have totally different arrangements.”* (ELE)

### Lack of Respect

Connected to the issue of a lack of financial compensation, the issue of a lack of respect for their work was brought up by some of the employees with lived experience. Some interviewees mentioned that the contributions of people with lived experience were not taken seriously by the other employees. For others, the lack of appreciation was shown by the insufficient provision of infrastructures necessary:

*“We are probably seen as patients who play around a bit and do their own thing without any real value.”* (ELE)

*“There is no internet. And if I want to do something, I want to do it properly. And I want it to be a success. That is why I sometimes continue to work at home and write there […] You cannot work properly in this office.”* (ELE)

Some interviewees saw a correlation between the lack of respect and institutional hierarchies. In contrast, others felt a lack of respect on the side of service users:

*“I think there is a hierarchy. […] We are guests, I do not think that we have a good reputation.”* (ELE)

*“Because some people look at me as if I were still a patient. And so, the way they talk to me is different than the way they speak with their therapist, for example […] less on the same eye-level.”* (ELE)

### Quality of Cooperation

The opinions of employees with lived experience varied in terms of their perception of the level and quality of collaboration between employees with and without lived experience. Some employees with lived experience described the collaboration as smooth, as they were allowed to make choices by themselves, giving them space to work independently and autonomously:

*“Everyone was very much on eye level. I never had the feeling that she was the boss or that she ordered me to do anything.”* (ELE)

*I can work pretty independently. Sure, there are some situations when you are being told to do something […].”* (ELE)

Other issues were voiced as well: power struggles, controversies regarding competencies required or responsibilities between employees with and without lived experience were reported. The fear of being replaced or giving up responsibilities was mentioned as a reason for this phenomenon:

*“What is the [peer] allowed to do? Is he allowed to have a key?”* (ELE)

*“Only 10% of the working hours may be done by peers […]. This shows that the [employees] are simply afraid of losing their jobs. And that could be prevented with new rules.”* (ELE)

Faced with this situation, employees with lived experience repeatedly felt that they were obliged to assert their own perspectives forcefully:

*“I often face the problem that when I get a certain impression of a patient and I mention this to the team […], [they] argue against it. And then I have to find a way […] to still assert myself.”* (ELE)

### Upsides and Downsides

At the same time, many employees with lived experience reported that they did benefit from the opportunity to work:

*“My job is fun. I have something to do. I could not imagine sitting at home all the time.”* (ELE)

*“My self-image became more positive. […] I have more self-worth. And I am not that sad any more about the fact that I'm not employable or cannot be placed on the regular job market.”* (ELE)

Because of their cooperation, employees with lived experience felt they were perceived more positively by the other employees than if they had simply remained a patient. This is seen as a helpful development in the process to break down the rigid boundaries between employees without lived experience on the one hand and employees with lived experience on the other hand:

*“I am really happy that I am not just perceived in my role as a patient, but also as someone who is engaged as a peer. This made a lot of things happen. Now I don't feel like I'm walking around being stigmatized as a patient.”* (ELE)

Yet, for some employees with lived experience, work was experienced as a burden and as demanding:

*“You hear a lot about the problems [with peer support]. And you know that on Monday, when you are back at your office, […] you will still be confronted with this.”* (ELE)

*“The weekend shifts are hard work. Patients in occupational therapy usually have drug issues. You have to be able to deal with them. Sometimes, there were fights […]. We even asked for help from the ward sometimes, right?”* (ELE)

In addition, difficulties setting boundaries were mentioned:

*“Setting boundaries is a big topic for me because I am very sensitive. I learned to turn my sensitivity into a strength. Ultimately, though, it is difficult for me to set boundaries.” (ELE)*

### Lack of Networking and Systematization of the Model

There is not a systematic network among the employees with lived experience. Some of the interviewees mentioned that the other employees without lived experience did not want this kind of exchange to occur:

*“The institutions do not want this, they don't want peers to sit together across the entire institution they are working in. Because they think that this automatically means that the institutions would be discredited.”* (ELE)

Still, some networks have been established independently or at a limited scale:

*“We are organizing ourselves. If that doesn't work, nothing will. The whole system will collapse.”* (ELE)

Despite the lack of systematic networking, some employees with lived experience perceive support among themselves as good:

*“It is a nice way of being together. Everything is very benevolent. It is a very compassionate group. We are all very different, but somehow it works. We like each other.”* (ELE)

Connected to this lack of networking is the criticism that the participation model in Geesthacht has not been planned systematically enough and needs further development. Defined structures with a clear and sufficient place for employees with lived experience have not been established yet:

*“That is the biggest mistake we have made. […] We did not structure the program. There is no institutional framework, there are too many loose ends.”* (E)

*“In the long run, I do see peers as set parts of the teams who can participate in supervision or in the planning of processes of change just as everyone else can.”* (E)

Furthermore, this lack of systematization requires a systematic evaluation of what has (and has not) been achieved:

“That we look at it: what was good, what do we want, what is not so good? What has just been tolerated? What do we want to support?” (E)

## Discussion

“Organic growth” t in Geesthacht over the last 13 years has led to the development of a care model that enables and promotes the participation of people with lived experience of crises and disabilities in the fields of work and activities. This care model aims at supporting people by fostering an active and autonomous life in their primary care setting right from the start, with the objective of making them less and less dependent on institutional support and in a sustainable way. These goals and principles regard the activities in Geesthacht in the field of work and employment, as described here in the manuscript, but also the different forms of assisted housing that have been established over years, combining aid from community services and volunteers with forms of home treatment, thereby enabling support in the services user's own environment and social interaction of people who are isolated.

The value of work, activities and/ or employment for the recovery of people with mental health crises and psychosocial disabilities has been demonstrated in various contributions and reviews (21–29). Work is a means for social integration and participation (23–25), it may have empowering effects (22, 26), and may contribute to developing a sense in one's existence (26, 27). Thus, enabling people to retain or gain work, activities and/ or employment has profound effects on many areas of life. Facing these benefits, it is a tragedy that unemployment and mental health disabilities are still strongly interrelated, leading to a high degree of personal and socioeconomic burden (30–34). The labor market still holds enormous barriers and access restrictions for people with psychosocial disabilities, the unemployment rate of this group of people is disproportionately high, as are their possibilities for societal participation.

In Germany, the mental health rehabilitation system is rather fragmented, consisting of numerous, discontinuous institutions and measures, often leading to a sense of confusion and lack of guidance on the side of users (33, 35, 36). Against this background, the Geesthacht is a rather unique attempt—at least in the German context—to both offer possibilities for work, activity, as well as participation and guidance on how to gradually progress within this system of support. Internationally, this model may be compared to other support systems, such as clubhouses (37), systems of supported employment (33, 38), or social firms (39, 40), allowing for gradual and often peer-based support for people with mental health crises and psychosocial disabilities. Yet, this paper focusing on the gradual development of the Geesthacht model over years as a response to the German-specific historical or contemporary health service and political developments, in what follows, a detailed comparison will be deliberately omitted in favor of a comprehensive discussion of its conditions of development as well as its inherent tensions and ambivalences.

### Conditions of Development

The model of participation in Geesthacht was developed through a stepwise process, leading to changes in institutional culture and its characteristics which thus vary depending on the institutions involved and the time. The fact that there was no central management of this process led to many instances of chaos and temporary (or long-lasting) solutions that are both satisfying as an achievement, and yet insufficient. However, the chaos involved can also be interpreted as a driving force and a necessary condition to enable a flexible, daily-life and person-centered model of support in Geesthacht. Thus, overall, we believe that the model can be seen as a constructive attempt to further de-institutionalize mental health care, with the focus on adapting psychiatric care to the lives of the people in care, instead of subordinating them to a rigid set of institutional rules, as is still the case in many institutions. The stated aim is to strengthen the autonomy and self-determination of the people who need support when experiencing mental crises and disabilities, which is certainly not always the case in Geesthacht, but has often been successful.

The development of the Geesthacht model started at a time when the principles enshrined in the CRPD, as well as the various critical approaches to the existing psychiatric care system were neither formulated nor well-known. As a result, it inheres conceptual tensions that will be further elaborated upon in the next subchapter of this discussion. The change of paradigmatic embedding of the psychiatric care system over the past 40 years has led to a progressive enrichment of concepts, initially drawing on ideas of the era of de-institutionalization, and gradually turning into more human rights-oriented models over the years. This evolution has resulted into a variety of co-existing work and support models that consequently draw on a wide spectrum of rationales, goals, resources, and equipment. Yet, despite these tensions, the Geesthacht model, from our perspective, is of contemporary importance as most people who experience mental crises and disabilities are still employed in sheltered workplaces (rather than supported employment), continue to live in asylums or residential care homes (instead receiving supported housing), or remain hospitalized (instead of receiving home treatment).

Fundamental to the changes in Geesthacht were adjustments to the budget allocation system for public hospitals. Care budgets previously based on either the number of days of care or the intensity of care were first supplemented with a regional mental health care budget in 2007, which was then encompassed in a global treatment budget from 2013 to 2019 (9). Thanks to flexibility allowed in terms of how the global budget was applied, it has been possible to establish a complex network of opportunities for participation and activities for people with lived experience. However, a certain mindset and certain principles of the people involved were equally important: leaving room for spontaneity and serendipity and for processes to develop, as well as the deep-seated conviction that participation, work, and activities are important existentially to all people, are only a few examples of an overall strongly ideological and value-based approach in Geesthacht. Without a strong philosophy, the project would not have evolved in this way.

### Tensions and Ambivalence

From our perspective, some of the benefits of the Geesthacht model of participation can be explained by the variety of tensions, ambivalence, and contradictions that it entails. As the research team, we found ourselves in varying emotional states that were subject to rapid change on several occasions. Feelings of enthusiasm, curiosity, skepticism, and sometimes discontent occurred in rapid succession.

Progressively, the divergence of perspectives of the different actors interviewed became apparent. While the statements made by those who were operational employees (with and without lived experience) were mainly related to their work tasks and the project which they evaluated critically, the executive staff (all without lived experience) tended to focus on the values and principles of this project, and did so in rather favorable ways. This difference represents one part of the enormous complexity and the contradictions that are characteristic of the Geesthacht model.

On the one hand, Geesthacht is characterized by a high willingness to take risks, to dare to try new things and to see that they are done, and to share responsibility. Employees with lived experience are trusted and given space to find their own way and shape their own lives. Instead of pre-planning or over-regulating participation and involvement in detail, opportunities for activities and work can grow organically between the parties involved, from concrete situations as well as from relationships that have grown over time. On the other hand, this approach has led to a lack of clear structures and responsibilities to date. Responsibilities often remain vague and there are no fixed formats for networking or supervision, leaving employees with lived experience sometimes alone with the question whom to approach.

Another area of conflict concerns the lack of a clear separation between therapy and work. It is possible for the service users in Geesthacht to participate early on during their hospital care by taking over tasks. The same or other activities may be continued after discharge and at times, may continue as paid employment. Many problematic situations are possible along the way, including the concealment of unpaid work. Yet, it remains a fact that for some of the employees with lived experience, no other avenue of participation or work would have been possible. Further, the employees with lived experience are involved as much as possible during these transitions between therapy and work: in each case they make decisions themselves or at least say they are ready to go along with them.

A third example of the many forms of ambivalence felt toward the Geesthacht model is the way in which the different roles change into another, which can be very challenging for all participants. The citation above from one of the employees with lived experience who continued her paid employment even at a time when she was experiencing a crisis illustrates these tensions. Thus, often it is impossible for persons to differentiate between their identity of service user and employee, a fact that involves both opportunities and risks. We were surprised that only a few persons interviewed evaluated these tensions negatively, suggesting that the demarcation between (“ill”) patients and (“healthy”) employees—a demarcation common in mental health care—played a minor role in Geesthacht.

### Seeing Mental Health Care as an On-Going Project

In summary, the question is whether more regulation in Geesthacht would have led to a better model. Clear rules and structures always involve the risk of establishing rigid roles and opportunities. In comparison, the flexible, vibrant, and open character of the Geesthacht model of participation was possible primarily because local structures and processes evolved in their own ways, with the responsibility to manage the various projects being left up to the participants themselves. Due to a variety of regulations and laws that have been implemented in recent years in Germany, such an approach has become less feasible in the inpatient sector, in spite of the fact that it has positive effects as demonstrated.

In this sense, mental health care is a field that benefits particularly if it is perceived to be an ongoing project that is constantly developing. A creative, evolving, and at the same time reliable environment is necessary to enable people (not only those experiencing a mental health crisis) to gain autonomy and to recover. This is documented in many histories of recovery and is also fundamental to the guiding principles of empowerment. Understanding mental health care as a project includes the possibility of trying things out (and failing), as well as providing a “responsive approach” (41), which is possible in Geesthacht right from the beginning of treatment. For the employees, tolerating risks and the willingness to take responsibility are substantial parts of the relationship and finding new methods with unknown outcomes is explicitly permitted.

The application of “mental health care as an on-going project” entails risks as well. If people are not able to communicate their needs, if power relations and dependencies are not dealt with transparently, if room for reflection is lacking, then those people who already have little influence in the care system may be the ones who continue to suffer. There is significant room for improvement regarding these issues remaining in Geesthacht as well. To what extent the openness and vibrancy of the project could be lost when dealing with these issues remains to be seen.

As described, the Geesthacht model also works well because many of the roles and opportunities for participation are not well-defined or clearly established. Therefore, it remains uncertain to what extent the employees with lived experience are actually working in accordance with the principles and methods of peer support (42). As mentioned above, some of these employees have received prior, formal trainings, some have not. Here too, no conclusions could be drawn from the interviews, despite the fact that the issue was addressed frequently. Indeed, it entails on the one hand, the danger of employees with lived experience being instrumentalized and asked to “toe the line,” but on the other hand, this loose framework also constitutes part of the spirit behind the project. Whether this ambiguity also represents added value in developing the field of peer support could be the subject of further research.

### Final Remarks

As explained above, the scope of this contribution allows us to cover in detail only those opportunities to participate in the fields of work and activities in Geesthacht. We have not reported here the significant results that we believe have also been achieved in the areas of participation in housing and treatment. In relation to the earlier question of whether the model described meets the standards of the CRPD, we will therefore only consider this area of participation in the areas of work and activity.

Article 27 of the UN CRPD recognizes the right for people with disabilities to work on an equal basis with others and under open, inclusive and fair conditions. This right also includes the opportunity to earn a living by work freely chosen and accepted, as well as the right to assistance in finding a job, starting a job, and returning to work (1). In this sense, people with lived experience of crises and disabilities in Geesthacht receive an extraordinary amount of support in the search for and participation in work and activities, in a field of care that - at least in Germany - is usually little concerned by these issues (17). At the same time, it depends on the individual case to what extent equal rights are applied in Geesthacht and how power imbalances play a role. Similarly, accessibility of the various options for participation, and to what extent work is compensated appropriately, differ between cases. Maybe it is precisely here that there is a clear need to catch up if the concepts of the CRPD are to be applied across the board.

Nevertheless, Geesthacht is a place where the boundaries between employees and service users are drawn less strictly. In the sense of an active community, relationships have been built up for years that transcend the traditional therapist and patient roles, which in many places continue to exist strongly and are very separate from one another. These relationships further enable, to varying degrees, a common sense of belonging in work and life. This is of great merit, and not only of analytical value and should serve as a model for the implementation of projects elsewhere.

In terms of compliance with the principles of the CRPD, the situation of (former) service users in Geesthacht regarding participation in work has improved considerably. This model has allowed them to become a substantial part of an activity-related and social network and, for some, to make their own living. On the other hand, the model in Geesthacht reveals the existing barriers to implementation of equal opportunities, even in the presence of strong motivation and the intense commitment of all participants. Many things remain to be done that must be initiated and managed structurally, as well as on the policy level.

## Data Availability Statement

The datasets underlying the current study are not publicly available due to the used data protection declaration and the nature of qualitative interviews where individual participants could be possibly identified. Parts of the dataset are available from the research group on reasonable request. Requests to access the datasets should be directed at Sebastian von Peter, sebastian.vonpeter@mhb-fontane.de.

## Ethics Statement

The studies involving human participants were reviewed and approved by Ethics Commission of Medical School Brandenburg Theodor Fontane; Institutional Ethics Committee of the Carl Gustav Carus University Dresden. The patients/participants provided their written informed consent to participate in this study.

## Author Contributions

SP and JS drafted the first version of the manuscript. SP was responsible for the background, result, and discussion section. JS drafted material and methods and developed tables, figures, and Supplementary Material. SP, JS, JZ, RG, BG, UG, AR, RB, and MH modified successive drafts. All authors were involved in preparing and conducting the present part of the PsychCare study, critically reviewed and commented on the manuscript, and read and approved the final version of the manuscript.

## Conflict of Interest

The authors declare that the research was conducted in the absence of any commercial or financial relationships that could be construed as a potential conflict of interest.

## Footnotes

^1^Beeker T, Von Peter S. Conducting a process evaluation coproduced by people with lived experience of mental distress – a metareflection and methodological concept. *Methodol Innovations*. (under review).

^2^Additional income is a capped amount that a person receiving a disability pension is allowed to receive for short-term or marginal employment.

^3^A mini-job is a marginal job with a maximum income of 450 Euros per month or short-term employment for a few weeks or months.
