# Supplementary material for: Supported Employment, Participation at Work, and Peer Support: A Qualitative, Participatory Case Study Report of the Geesthacht Model
Source: Front Psychiatry. 2021 Apr 23;12:634080. doi: 10.3389/fpsyt.2021.634080 (PMC8102772; doi:10.3389/fpsyt.2021.634080)
Supplement: Supplementary file 1 [file Table_1.DOCX]

***Supplementary Material***

**Table S1.** Overview of stakeholders and institutions, fields of work, funding, and work locations

| **Stakeholder/ Institution** | **Fields of work** | **Work tasks of Peers** | **Funding sources/work or employment agreements** | **Work location** |
| --- | --- | --- | --- | --- |
| “Arbeit nach Maß”  (NGO that arranges work tailored to the person’s needs) | Logistics  Transportation  Social work  Peer Support  Catering | Maintenance of hospital beds  Service user shuttle  Peer Support  Contacts and meetings | Funding sources: IA, PB, occupational therapy, global budget (in the past)  Work agreements: additional earnings, “mini-job”, paid employment, expense allowance, “incentive pay” | Clinic  Home environment  Outpatient occupational therapy centers  ABC team (clinic)  Laundromat café  Art Gallery |
| “Pflege nach Maß” (NGO that arranges mental health care tailored to the person’s needs) | Care  Peer support | Home care  Peer support | Funding sources: Home care (§37 SGB V), PB, global budget (in the past)  Work agreements: additional earnings, “mini-job”, paid employment, expense allowance, “incentive pay” | Home care groups  Home environment (HT) |
| “Wohnen nach Maß” (NGO that arranges housing tailored to the person’s needs) | Housing  Crisis respite | Housing therapy/housing first  Rooms for crisis respite | Funding sources: housing allowance, hospital earnings from rental accommodations, IA, Care (SGB XI, V)  Work agreements: mini-jobs, provision of housing, paid employment | Home environment  (apartment/house) |
| ABC-team (+ Tuesday group as the central hub) | Social work  Peer-support  Non-professional help | Assistance by peers to complete gov. and social service admin. obligations  Weekend shifts at the day hospital  Peer support  User support & guidance | Funding sources: PB, Occupational therapy, global budget (in the past), IA  Work agreements: Volunteering, volunteer allowances, mini-job/additional earnings, paid employment | Clinic  Day hospital  Outpatient occupational therapy centers  Home environment |
| Outpatient occupational therapy center  “Mucha” and “Ergoaktiv e.V.” | Peer support  Social work  Occupational therapy | Peer support in the psychosomatic ward  Assistance to ABC-team | Funding sources: Occupational therapy, global budget (in the past), PB  Work agreements: mini- job/ additional earnings, expense allowance, incentive pay | Clinic (“buys” hours)  ABC-team (clinic)  Outpatient occupational therapy centers |
| Outpatient occupational therapy center “Huth” | Peer-Support  Occupational therapy | User support & guidance  Group work  Occupational therapy related tasks | Funding sources: Occupational therapy, model budget (in the past), PB  Work agreement: Paid employment (max. 10% of all paid employment) | Outpatient occupational therapy centers |
| Crisis respite houses/ apartments | Housing  Peer support  Daily life support | Daily life and housing support  Peer support  Assistance | Funding sources: Funding from special needs housing, long-term care insurance, PB, global budget in the past  Work agreements: additional earnings, mini-job, paid employment, expense allowance, incentive pay | Various apartments and buildings |
| Laundromat café | Peer support  Catering  Scheduling and communication | Contacts and meetings  Instruction and consulting  Work therapy  Home treatment | Funding sources: IA, PB, global budget (in the past), occupational therapy (in the past)  Work agreements: additional earnings, mini-job, paid employment, expense allowance, incentive pay | Laundromat café |
| Akron association | Peer Support  Budget assistance | Non-professional aid at various sites  Peer work funded by the PB  Agricultural work (in the past)/ work therapy | PB as payment for peers and non-professionals | Flexible locations  Agricultural projects (in the past) |
| Additional offers and cooperating institutions (among others):   - Workers Welfare Association - Social shopping mall - Second-hand shops - Individual case support (Brücke) |  |  |  |  |

*Legend: PB = Personal Budget, HT = Home-Treatment, IA = Integration Assistance. The clinic itself is not mentioned as it does not act as an employer of peer employees.*

**Table S2.** Interview guide

| **Introductory questions** | **Further questions to probe** |
| --- | --- |
| How exactly does the system of “stepwise integration” in Geesthacht work? | What steps/ modules etc. are usually planned? Is it possible to systematize these?  How are peers introduced into peer work? How do service users become volunteers? How do volunteers become paid employees?  How are job positions advertised? Are there criteria regarding the eligibility of former service users to become peers? How are people matched to different teams? Who decides which former service users become active as peers? How are the arrangements made?  How many peers (exact number) perform assistance and care-related work and in what areas (including over the years and in the past)? Are there any factors influencing this? How many peers volunteer (unpaid work), how many have paid employment? What is the ratio between peers and professionals per team? |
| What are the necessary conditions and preparations at the institutions to integrate such a model? | What barriers exist to integration of peers? On the side of the peers, on the side of the professionals? Barriers due to structural reasons? What barriers are created by the vague occupational status of peers?  Were there any pilot projects that served as a model? Or did you just try things out? Were there any similar projects that failed and, if yes, why?  How are the peers paid? What is the rationale behind these forms of payment? What is the legal framework/method of funding behind such compensation? How are the various forms of funding combined with each other?  **Expertise**  What qualifications do (did) the peers have (before starting to work as a peer)? How do these qualifications play a role in their work as a peer? How are they integrated in the job? By whom (by peers or professionals; are new professionals also supported by peers)?  What further training did the peers receive during their employment? Did peers and professionals together participate in on-going training? Were there any advanced training sessions or supervision?  **Problem-solving strategies**  Is there a mentor program? If yes, how does it work? Is there common or separate peer consulting/supervision? How are conflicts within the team reflected and solutions found? How open and transparent is this process?  **Work burden and potential burnout**  How do employees take care of their own mental health and well-being in general? What happens in case of a heavy workload (e.g. many service users with a great need of support at short notice)? How is the work distributed? How are different levels of resilience and readiness to work dealt with?  What is the approach if an employee has a personal crisis? What is the approach if a peer worker experiences a crisis? How are the work burden, personal limits, and barriers dealt with? How do people speak about it? How do the peers respond? How do the other employees respond?  Who can mention something if he/she suspects that an employee is in crisis? How does it work? Are employees in crisis able to use the services of the clinic and the system? (How does that change the collaboration afterwards?) (How is this communicated?) Were differences observed regarding the sick leaves of the various groups of employees?  **Questions regarding the culture of participation**  Were peers involved in the concept and planning of the services and the regional model? If yes, how? How are peers involved in hiring new employees? Are there opportunities for advancement for peers or do they hit a glass ceiling?  Were peers involved in the development of the peer model (e.g. job descriptions)? How are peers involved in evaluation and development of this model? |
| What do the peers do in the facilities? | Where are they employed? To what extent are peers employed? What tasks do they carry out? What tasks are only carried out by professional employees? Are there general rules (e.g. peer/service user ratio is higher than professional/service user ratio or vice-versa)? Are there individual regulations (e.g. only a specific person performs specific tasks)?  How are jobs with job descriptions advertised? What tasks and activities are appropriate/ inappropriate? Why? Who decides on whether they are appropriate? What skills/experiences do the peers add currently to the services? What roles and tasks are experienced as being helpful?  How do peers implement their specific experiential knowledge? Did they develop specific services? Did they develop specific methods regarding how to deal with certain situations or needs?  How is information exchanged regarding work/task distribution and the different roles the peers can and would like to take on? How are different levels of authority, responsibility, liability regarding decisions to accompany users dealt with? |
| What models of work and participation exist peer-to-peer and peer-to-professional respectively? | Which proved successful? How are these models experienced? What are the characteristics of the relationships between peer/peer, professional/professional, peer/professional as colleagues?  What do professionals expect from and think about the peers and their expertise? What roles should the peers play/not play? What roles do they want/not want to play? What reservations do professionals have about peers? What happens in teams if employees do not meet others’ expectations? How do you deal with it?  How are different positions, perspectives regarding the support to be given to service users dealt with? How are different personal abilities and possibilities of employees respected in their daily work?  What is the impact of the role change from service user to employee for peers? What is the impact of the role change from therapist to colleague for professionals? How do the transitions between volunteering and paid employment go for peers? What is the effect of the different financial compensation on cooperation? How is the financial compensation perceived?  How do peers and professionals deal with (overly) high expectations of their services? How do peers deal with the pressure that is created by the feeling that they need to show commitment (and stable health) to be accepted as an employee? |
| What effects does the model have on the teams and the peers? | **Effects on the teams**  How does the employment of peers affect the teams? What aspects of the teamwork changed? What perspectives/beliefs of the professionals regarding people in crisis and how to handle the situation were changed? What is the impact of employment of peers on practice?  What changes were particularly surprising? What changes were burdensome/relieving? What changes were permanently established? Did changes in the daily work routine occur that were not understandable or not justifiable for professionals/ peers? Which ones? How were they dealt with?  What are the differences between the aims of peers and professionals regarding their work with service users? (What are the goals of their work with service users?)  What do the teams want in the future? What do they want to change, what are their worries and hopes?  **Effects on the peers**  How did the perspectives/beliefs of peers change as cooperation developed over time? How did the method and approach to support to service users change over time? What circumstances led to this?  Is the mainstreaming/appropriation of peer support being prevented in Geesthacht? What is being done to preserve the specific characteristics of their role/value?  Were there any tasks that peers had to do that were against their beliefs? Which ones? How was this dealt with?  What do the peers want in the future? What do they want to change, what are their worries and hopes? |
